# Digestate from Agricultural Biogas Plants as a Reservoir of Antimicrobials and Antibiotic Resistance Genes—Implications for the Environment

**DOI:** 10.3390/ijerph20032672

**Published:** 2023-02-02

**Authors:** Izabela Wolak, Sylwia Bajkacz, Monika Harnisz, Klaudia Stando, Magdalena Męcik, Ewa Korzeniewska

**Affiliations:** 1 Department of Water Protection Engineering and Environmental Microbiology, Faculty of Geoengineering, University of Warmia and Mazury in Olsztyn, Prawocheńskiego 1, 10-720 Olsztyn, Poland; 2 Department of Environmental Biotechnology, Faculty of Energy and Environmental Engineering, Silesian University of Technology, Akademicka 2, 44-100 Gliwice, Poland

**Keywords:** ARGs, heavy metals, sewage sludge, slaughterhouse waste, cattle manure, maize silage

## Abstract

Antimicrobials and antibiotic resistance genes (ARGs) in substrates processed during anaerobic digestion in agricultural biogas plants (BPs) can reach the digestate (_D_), which is used as fertilizer. Antimicrobials and ARGs can be transferred to agricultural land, which increases their concentrations in the environment. The concentrations of 13 antibiotics in digestate samples from biogas plants (BPs) were investigated in this study. The abundance of ARGs encoding resistance to beta-lactams, tetracyclines, sulfonamides, fluoroquinolones, macrolide-lincosamide-streptogramin antibiotics, and the integrase genes were determined in the analyzed samples. The presence of cadmium, lead, nickel, chromium, zinc, and mercury was also examined. Antimicrobials were not eliminated during anaerobic digestion. Their concentrations differed in digestates obtained from different substrates and in liquid and solid fractions (ranging from 62.8 ng/g clarithromycin in the solid fraction of sewage sludge digestate to 1555.9 ng/L doxycycline in the liquid fraction of cattle manure digestate). Digestates obtained from plant-based substrates were characterized by high concentrations of ARGs (ranging from 5.73 × 10^2^ copies/g_D_ *cfx*A to 2.98 × 10^9^ copies/g_D_ *sul*1). The samples also contained mercury (0.5 mg/kg dry mass (dm)) and zinc (830 mg/kg dm). The results confirmed that digestate is a reservoir of ARGs (5.73 × 10^2^ to 8.89 × 10^10^ copies/g_D_) and heavy metals (HMs). In addition, high concentrations of integrase genes (10^5^ to 10^7^ copies/g_D_) in the samples indicate that mobile genetic elements may be involved in the spread of antibiotic resistance. The study suggested that the risk of soil contamination with antibiotics, HMs, and ARGs is high in farms where digestate is used as fertilizer.

## 1. Introduction

Due to growing levels of waste generation around the world and a higher demand for alternative and environmentally friendly energy, anaerobic digestion (AD) is becoming an increasingly popular method of stabilizing various types of organic matter substrates [1]. The popularity of installations such as biogas plants (BPs) is on the rise in Europe, including in Poland, and the number of BPs continues to increase. A total of 132,000 engineering biogas projects have been implemented around the world, including 17,783 in Europe. Biogas is upgraded to biomethane in 700 plants worldwide, 540 of which are located in Europe [2,3]. In Poland, the first BP was commissioned for use in 2005, and there were 128 agricultural BPs at the end of 2021 [4].

Most BPs use manure [5], sewage sludge [6], slaughterhouse waste [7], meat and bone meal [8], ensilaged biomass such as maize silage [9] and sugar beet silage [10], potato pulp [11], and confectionery press cake [12] as substrates for AD. These substrates are processed into nutrient-rich digestate. Digestate contains organic and mineral compounds, nitrogen, macronutrients, and micronutrients, and it is colonized by digestate-specific microbiota composed of diverse microbial communities which participate in AD [13]. For many years, digestate has been used mainly as agricultural fertilizer around the world [14]. Digestate is a rich source of nutrients that promote plant growth, but it also contains antibiotics and their metabolites, antibiotic-resistant bacteria (ARB), antibiotic resistance genes (ARGs), and heavy metals (HMs). Therefore, there is a high risk that these micropollutants will be transmitted to the soil when digestate is used as organic fertilizer [15].

Most antimicrobials, ARB, ARGs, and HMs present in digestate originate from substrates that are used in the AD process. Sewage sludge, the byproduct of wastewater processing in wastewater treatment plants (WWTPs), is also used as feedstock in BPs, and it contains large quantities of drugs, ARB, ARGs, and HMs [13] because most pharmaceuticals are not fully metabolized in the human body and are evacuated to WWTPs with wastewater. Moreover, HMs also reach WWTPs with industrial wastewater and manure, and they contribute to environmental pollution and can participate in the transmission of ARGs. Heavy metals such as cadmium (Cd), mercury (Hg), nickel (Ni), chromium (Cr), and lead (Pb) are characterized by high biostability and a long half-life in the environment, and they can also exert selection pressure on microorganisms [16]. Sewage sludge produced during wastewater treatment is used as a substrate for AD in agricultural BPs, often in combination with other agricultural feedstocks.

In digestate, pharmaceuticals and HMs can also originate from manure and agricultural wastes that are rich sources of mineral substances and microorganisms and are most widely used as substrates in AD. According to the literature, global annual antibiotic consumption in livestock production has been estimated at 172 mg/kg in pigs, 148 mg/kg in chickens, and 45 mg/kg in cattle [14]. In animals treated with antibiotics, 30% to even 90% of antimicrobials and their metabolites are retained in urine and feces, and thousands of tons of drugs are evacuated to the environment with manure [14,15]. In China, the world’s largest meat producer, 45,000 tons of antibiotics were excreted with animal feces in 2013 [14].

Drugs that are introduced to the environment with digestate-based fertilizers can be highly toxic, and they can cause selection pressure which increases the concentrations of ARGs in soil and contributes to antibiotic resistance (AR) [9]. In addition, no distinction between human and veterinary medicinal products is made in Europe; therefore, the same antimicrobials or drugs belonging to the same class are used in human and veterinary medicine. For example, fluoroquinolones and sulfonamides are classified as veterinary drugs in China, but they account for 2.2% and 9.2%, respectively, of all antimicrobials sold in Europe, and are used in both human and veterinary medicine. Beta-lactams and tetracyclines are also widely used in human and veterinary medicine in Europe [17,18,19]. Macrolides [20], lincosamides [21], and dihydrofolate reductase inhibitors (trimethoprim) [22] have also been identified in agri-food products. Due to the wide availability of pharmaceutical products on the market, pathogens that are resistant to a single drug can develop resistance to an entire class of related antimicrobials [23,24].

The rate at which chemotherapeutics and HMs are degraded in soil is determined by temperature, soil texture, organic carbon content, and the resistance profile of soil-dwelling microorganisms [14]. Pharmaceuticals accumulated in soil can be carried by surface runoffs to water bodies which are used in livestock production [15], and they can be ingested by animals in successive stages of the food chain. These drugs can then be transmitted to humans via contaminated animal-based products [16]. Fresh produce can be a source of clinically significant drug residues, ARB, and ARGs for consumers [25]. Antibiotic-resistant bacteria pose a significant risk of infection, and the therapeutic efficacy of antimicrobials is being compromised by growing levels of AR in humans and animals.

Antibiotics and HMs present in the environment can contribute to the spread of ARGs and ARB in soil, and they can trigger the emergence of new ARGs or increase their concentrations in a given environment [16]. Antimicrobial resistance genes can also spread rapidly between microbial communities via horizontal gene transfer (HGT) because microorganisms present in substrates subjected to AD and digestate can harbor mobile genetic elements (MGEs) such as integrons [17]. For this reason, the concentrations and spread of antibiotics, HMs, and ARGs in digestate produced by agricultural BPs should be monitored to prevent or limit the transmission of ARB and ARGs in the environment, and to minimize selection pressure on environmental microorganisms.

The digestate can be considered a rich source of valuable minerals, but at the same time, unfortunately, it can also be a reservoir of antibiotics, ARGs and heavy metals for plants and microorganisms. There is a large research gap in the literature related to this topic, because there are still only few data on the content of ARGs, HMs, and pharmaceuticals and their persistence in the fermentation mass based on various substrates, which during soil fertilization with digestate can significantly affect their content in soil and edible vegetables. So far, there are no studies available with the content of so many antibiotic groups, HMs, and ARGs in digestate based on different substrates and the possible environmental implications for the soil–plant–animal–human system related to their transfer to the soil along with digestate used as fertilizer. Therefore, in this preliminary study, digestate samples from agricultural BPs stocked with various substrates, including sewage sludge, animal manure, and green waste, were analyzed for the presence of beta-lactam (amoxicillin, ampicillin), tetracycline (tetracycline, oxytetracycline, doxycycline, chlortetracycline), sulfonamide (sulfamethoxazole, sulfonamide), fluoroquinolone (enrofloxacin, ciprofloxacin), macrolide (clarithromycin), lincosamide (clindamycin) antibiotics, and dihydrofolate reductase inhibitors (trimethoprim). Digestate samples were also examined for the presence of HMs such as Cd, Pb, Ni, Cr, zinc (Zn), and Hg, as well as genes encoding resistance to beta-lactams (*bla*_TEM_, *bla*_OXA_, *cfx*A), tetracyclines (*tet*A, *tet*M), sulfonamides (*sul*1, *sul*2), fluoroquinolones (*aac 6′-Ib-cr*, *qep*A), and macrolide-lincosamide-streptogramin (MLS) class antibiotics (*erm*F). The prevalence of class 1 and 2 integrase genes (*intI*1 and *intI*2) was also determined in digestate samples. The environmental implications of uncontrolled transmission of drugs and ARGs to agricultural land were assessed.

## 2. Materials and Methods

### 2.1. Characteristics of Biogas Plants and Digestate Samples

Three fully operational agricultural BPs in Poland were selected for the study and labeled as BP1, BP2, and BP3. The selected BPs process various types of substrates whose characteristics and composition are presented in Table 1.

Digestate samples collected from each BP were labeled with the number of the corresponding BP and the sampling season (S—spring, W—winter): BP1S, BP1W, BP2S, BP2W, BP3S, and BP3W.

The presence and concentration of drugs and HMs, as well as the presence and abundance of ARGs, were examined depending on the substrate composition of digestate samples. Digestates were sampled in winter and spring, which differ in the availability of various substrates and weather conditions (such as temperature) and are characterized by increased disease incidence and, consequently, antibiotic consumption. Digestate samples were collected directly from the outlets of full-scale bioreactors operating under mesophilic conditions. Digestate produced by all three BPs is used as organic fertilizer in agricultural production.

### 2.2. Determination of Antibiotics in Digestate Samples

The analytical procedure for identifying antibiotics in the preliminary study was based on the methods that had been used in our previous research [26,27,28]. Digestate samples were analyzed for the presence of the following antibiotics according to the procedure presented in Table S1: amoxicillin—AMO, ampicillin—AMP, tetracycline—TC, oxytetracycline—OXY, doxycycline—DOX, chlortetracycline—CHLOR, sulfamethoxazole—SMX, sulfonamide—SFD, enrofloxacin—ENF, ciprofloxacin—CIP, clarithromycin—CLR, clindamycin—CLD, and trimethoprim—TRI.

The limit of quantification (LOQ) was set at 0.1 ng/L of fresh liquid digestate fraction and 0.1 ng/g of fresh solid digestate fraction.

### 2.3. Determination of HMs in Digestate Samples

The presence and concentrations of six HMs, i.e., Cd, Pb, Ni, Cr, Zn, and Hg in digestate samples were determined by inductively coupled plasma optical emission spectroscopy (ICP-OES). The analyses of digestate samples were conducted in an accredited laboratory of the Institute for Ecology of Industrial Areas (IETU), Katowice, Poland [29,30].

### 2.4. gDNA Isolation

Digestate samples (2 g) were placed in 2 mL Eppendorf tubes and centrifuged at 8000 rpm for 10 min. The supernatant was discarded after centrifugation. Genomic DNA (gDNA) was isolated from the pellet in triplicate, according to the instructions provided by the manufacturer of the Fast DNA Spin Kit for Soil^®^ (MP Biomedicals™, Carlsbad, CA, USA). The concentration and quality of the isolated gDNA were evaluated with the Multiskan Sky spectrophotometer (Thermo Scientific™, Waltham, MA, USA). Additionally, gDNA was isolated in triplicate from each sample, and the isolates were pooled to obtain a representative gDNA sample for each digestate sample. The samples were freeze-stored (−20 °C) until analysis.

### 2.5. Quantification of ARGs and Integrase Genes

The presence and abundance of genes encoding resistance to beta-lactams (*bla*_TEM_, *bla*_OXA_, *cfx*A), tetracyclines (*tet*A, *tet*M), sulfonamides (*sul*1, *sul*2), fluoroquinolones (*aac 6′-Ib-cr*, *qep*A), and macrolide-lincosamide-streptogramin (MLS) class antibiotics (*erm*F) were analyzed in digestate samples. The prevalence of class 1 and 2 integrase genes (*intI*1 and *intI*2) was also determined. Genes conferring resistance to the above classes of antibiotics were analyzed because these antimicrobials are widely used in agriculture and in human and veterinary medicine. The presence and abundance of integrase genes were examined to assess the degree to which MGEs participate in the spread of AR. Antibiotic resistance genes were quantified in the quantitative Polymerase Chain Reaction (qPCR) in the LightCycler^®^ system (Roche Diagnostics, Mannheim, Germany). The SYBR Green dye (Roche Diagnostics, Mannheim, Germany) was used in the reaction mixture with a total volume of 15 µL. The qPCR mix contained 0.8 µL of the DNA matrix, 0.4 μL of the corresponding primer with a concentration of 10 μM, 7.5 μL of SYBR Green, and 5.9 μL of RNase-free water. Genes were amplified in triplicate, and amplification parameters are presented in Table S2 (Supplementary Materials). Melt curves were generated and analyzed for non-specific amplification. Fluorescence excitation spectra were analyzed with the use of LightCycler^®^ v. software (version 1.5.0). The number of gene copies was expressed in terms of absolute abundance (AA) in 1 g of digestate (ARG copy number/g_D_).

### 2.6. Statistical Analysis

Significant differences between ARG and HM concentrations in samples were determined in the Kruskal–Wallis non-parametric test (Statistica v. 13.3). The correlations between ARG abundance, antibiotic concentrations, and HM concentrations in samples were determined in the Principal Component Analysis (PCA). Principal Component Analysis was applied using as variables all data from samplings BP1S, BP1W, BP2S, BP2W, BP3S, and BP3W regarding the concentration of 13 antibiotics such as AMO, AMP, TC, OXY, DOX, CHLOR, SMX, SFD, ENF, CIP, CLR, CLD, and TRI; ARGs concentration, such as *bla*TEM, *bla*OXA, *cfx*A, *tet*A, *tet*M, *sul*1, *sul*2, *aac 6′-Ib-cr*, *qep*A, *erm*F; and the integrase genes concentration (*intI*1 and *intI*2) and HMs concentration such as Cd, Pb, Ni, Cr, Zn, and Hg. Moreover, non-parametric Spearman’s correlation analysis was performed to indicate the effect of antibiotic concentration on the ARGs and integrase genes studied. The results were regarded as statistically significant at *p* < 0.05. The results of statistical analyses were visualized with the use of GraphPad Prism 8.4 software (v. 9.5.0).

## 3. Results and Discussion

### 3.1. Presence and Concentrations of Antibiotics in Digestate Samples

**Figure 1:**
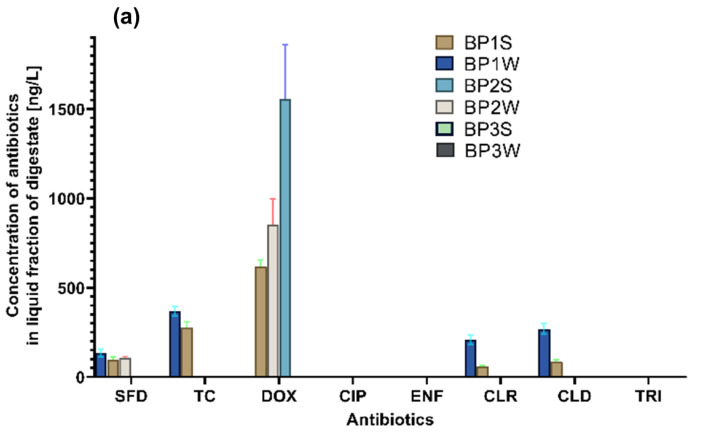

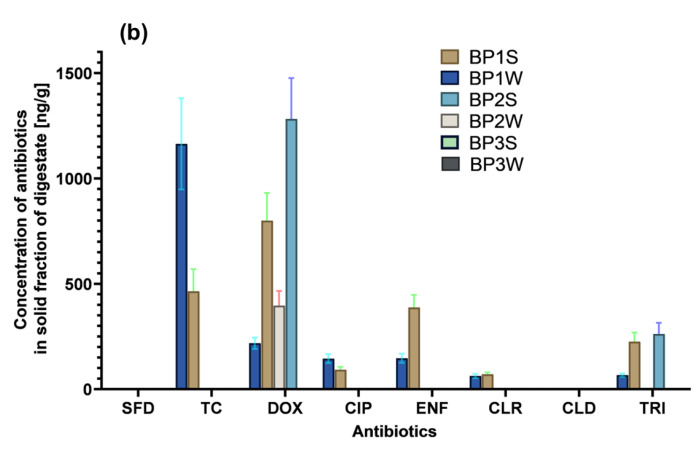
Concentrations of antibiotics in (**a**) liquid and (**b**) solid fractions of digestate. Error bars denote standard deviation (SD).

The study revealed that 8 out of the 13 tested antibiotics were present in digestate samples. The presence of pharmaceuticals was determined by the composition of the examined digestates. Digestate samples differed significantly in antibiotic concentrations (Figure 1; Table S3, Supplementary Materials) (Kruskal-Wallis, ANOVA, *p* < 0.05). None of the examined digestate samples contained SMX, OXY, CHLOR, AMO, or AMP.

The abundance of antibiotics was highest in samples BP1S and BP1W from the BP stocked with sewage sludge. The concentrations of drugs belonging to each antibiotic class also differed considerably between the samples of sewage sludge digestate. Moreover, liquid and solid fractions of digestate also differed in terms of the presence and concentrations of the analyzed drugs. Antibiotics were identified more often and with similar frequency in the solid fraction. The presence of drugs in sewage sludge digestate could be attributed to substrate contamination before AD. Antibiotics are widely prescribed around the world, and they are not fully metabolized in the body, which is why high concentrations of pharmaceuticals are present in sewage sludge. Moreover, according to the literature, the incidence of acute respiratory infections varies on a seasonal basis [31], which is why drug consumption and transmission to WWTPs with wastewater is intensified in selected seasons of the year [32]. Therefore, significant quantities of antimicrobials that are not biodegraded in the wastewater treatment process can be carried to sewage sludge, which is used as a substrate in bioreactors, which explains their presence in the examined digestate samples. It should also be noted that pharmaceuticals including antibiotics are often overused and used without medical guidance, which can significantly increase their concentrations in sewage sludge [33,34].

#### 3.1.1. Tetracyclines

The analyzed digestate samples contained TC and DOX antibiotics from the tetracycline class. Tetracycline was identified in samples BP1W and BP1S, in both liquid and solid fractions of the examined matrix. However, TC concentrations differed significantly (Kruskal–Wallis, ANOVA, *p* < 0.05) between liquid and solid fractions. In sample BP1W, TC concentration was determined at 1164.4 ng/g in the solid fraction and 368.7 ng/L in the liquid fraction. In sample BP1S, TC concentration was determined at 464.8 ng/g in the solid fraction and 275.6 ng/L in the liquid fraction.

Significant differences in DOX concentrations were observed between digestate samples, depending on their substrate composition (Kruskal–Wallis, ANOVA, *p* < 0.05). Doxycycline was identified in the samples collected from two out of the three analyzed BPs (samples BP1W, BP1S, BP2W, and BP2S). Samples BP1W and BP1S were obtained from a BP processing sewage sludge, whereas samples BP2W and BP2S were collected in a bioreactor stocked with cattle manure and maize silage. Doxycycline was identified in nearly all of the above samples, in both solid and liquid fractions. Only the liquid fraction of sample BP1W was free of DOX. In the solid fractions of samples BP1W, BP1S, BP2W and BP2S, DOX concentration ranged from 218.1 ng/g to even 1282.5 ng/g. In the liquid fractions of samples BP1S, BP2W and BP2S, DOX concentration was determined in the range of 620.4 ng/L to 1555.9 ng/L. In the studied digestate samples, DOX was present in higher concentrations than the remaining antibiotics. Doxycycline levels were influenced by the substrate composition of digestate samples, but not by digestate fraction (solid or liquid).

Similar results were reported by other authors [35] who identified DOX in digestate samples and found that the half-life of DOX in digestate can be as long as 91 days. In another experiment [36], the concentration of TC reached up to 36 μg/L in both liquid and solid digestate fractions. The results of this study and the findings of other authors indicate that tetracycline class drugs are not fully removed in the AD process, and that repeated soil fertilization with digestate containing tetracyclines can lead to their accumulation in soil and can directly affect the soil microbiome and resistome [37].

#### 3.1.2. Sulfonamides

Significant differences in the presence and concentrations of SFD were noted in solid and liquid fractions of digestate samples (Kruskal–Wallis, ANOVA, *p* < 0.05). Sulfonamides were identified in liquid fractions of samples BP1W, BP1S, and BP2W (Kruskal–Wallis, ANOVA, *p* < 0.05). Samples BP1W and BP1S were obtained from a BP processing sewage sludge, whereas the sample BP2W was collected from a bioreactor stocked with cattle manure. Sulfonamide concentrations ranged from 98.2 ng/L to 136.5 ng/L. According to other authors, SFD are effectively biodegraded during AD, but their removal rate can be affected by the conditions inside the bioreactor, such as the presence of iron, sulfates, and methane-producing microorganisms [16,17]. The biodegradation of SFD during AD can be attributed to the presence of –NH_2_ and –CH_3_ electron donating groups in the drugs’ molecular structures. The biotransformation of SFD during AD can lead to the reduction in the electron-withdrawing sulfonyl group [16] or the iron- and sulfate-catalyzed cleavage of the isoxazole ring [17]. Interestingly, in the present study, SFD were identified in the liquid, but not in the solid phase of digestate. According to many authors, SFD are biodegraded more rapidly in the liquid fraction, but this class of antibiotics could have been more effectively eliminated from the solid fraction due to the accumulation of chemical compounds that promote their biodegradation, whereas in the liquid fraction, these compounds were diluted, which could have affected their biodegradation pathway [38]. The SFD biodegradation pathway could have also been influenced by the presence of iron and sulfates in digestate, which is why this drug was not identified in half of the examined digestate samples.

Similar results were reported in other studies [20]. Numerous authors identified SFD in digestate obtained under laboratory conditions [18] and in digestate from industrial bioreactors [19]. In the present study, SFD concentrations were highest in sewage sludge and cattle manure digestates. These observations can probably be attributed to direct consumption of SFD by patients and increased use of SFD in livestock production. Sulfonamides are broad-spectrum antibiotics that are widely used in veterinary medicine, and they can significantly contribute to the spread of AR in the environment due to their high mobility and low susceptibility to biodegradation [39]. For example, pharmaceuticals introduced to soil with digestate can directly affect microbial communities, reduce bacterial groups sensitive to chemotherapeutics, and contribute to AR. Drugs can also exert a negative impact on crops by promoting the spread of ARGs in edible plant parts [28] and by inhibiting plant growth [40].

#### 3.1.3. Fluoroquinolones

Ciprofloxacin was identified in samples BP1W and BP1S collected from a BP stocked with sewage sludge. Ciprofloxacin concentration was significantly higher in the solid fraction of sample BP1W (145.3 ng/g) than sample BP1S (92.4 ng/g) (Kruskal–Wallis, ANOVA, *p* < 0.05). This drug was not identified in the liquid fraction. Enrofloxacin was detected only in the solid fraction, and similarly to CIP, it was identified in samples BP1W and BP1S (digested sewage sludge) at concentrations of 147.1 and 387.9 ng/g, respectively (Kruskal–Wallis, ANOVA, *p* < 0.05).

In an Austrian study, antibiotics were identified in digestate samples, and digestate-fertilized soils were contaminated with ENF and CIP [41]. According to the literature, fluoroquinolones are easily adsorbed on various types of organic substrates [42] during AD. These observations indicate that ENF and CIP pose a considerable risk of ecotoxicity when introduced to soil with digestate. These drugs can exert selection pressure on soil-dwelling microorganisms and contribute to the spread of ARGs and AR in the soil microbiome [43].

#### 3.1.4. Macrolides and Lincosamides

In sewage sludge digestate (BP1W), CLR and CLD concentrations were significantly higher (more than three times) in the liquid fraction (208.9 and 268.4 ng/L, respectively) than in the liquid fraction (56.97 ng/L and 88.6 ng/L, respectively) of sewage sludge digestate (BP1S) (Kruskal–Wallis, ANOVA, *p* < 0.05).

Other studies [44,45,46] revealed that CLR and CLD were adsorbed by sewage sludge, which indicates that the use of sewage sludge as fertilizer, even after AD, without further processing can be a source of soil and groundwater contamination with antibiotics and ARGs. This is an important observation in the context of European and Polish directives which regulate only the content of nutrients such as nitrogen in organic fertilizers, but not the content of antibiotics which reach the soil with fertilizers from processed agricultural waste [47]. The above increases the risk that significant quantities of pharmaceuticals will be introduced the environment in an uncontrolled manner with digestate-based fertilizers.

#### 3.1.5. Trimethoprim

Trimethoprim was identified in the solid fraction of samples BP1W and BP1S at concentrations of 66.8 ng/g and 226.1 ng/g, and in the solid fraction of sample BP2S (cattle manure and maize silage digestate) at 261.9 ng/g (Kruskal–Wallis, ANOVA, *p* < 0.05). Trimethoprim contains two aromatic rings, a double aminated pyrimidine ring and phenyl-trimethyl ether. According to the literature, this drug is effectively biodegraded (in more than 80%) in both liquid and solid fractions of organic matter [48]. Anaerobic biodegradation of TRI can be catalyzed by the cleavage of phenyl methyl ether or the substitution of the pyrimidine ring, depending on AD conditions inside the bioreactor [49]. Trimethoprim contains only functional electron donating groups, such as –NH_2_, –OH, –CH_3_ and –OCH_3_, in its molecular structure, which increases its susceptibility to biodegradation. Moreover, the drug has different biodegradation pathways under anaerobic conditions, depending on the availability of electron acceptors, both inorganic (such as NO_3_^−^, SO_4_^2−^, Fe^3+^, and Mn^4+^) and organic (such as sulfoxide and dimethyl sulfide) [50]. The presence of TRI in both sewage sludge and manure digestates suggests that this drug had been administered to human and veterinary patients in seasons with increased disease prevalence. In human medicine, TRI is a first-line antibiotic in the treatment and prevention of urinary tract infections, and it is used as a prophylactic in veterinary medicine and livestock production. When administered frequently, this drug can be transferred to sewage sludge and cattle manure which are used as feedstocks in agricultural BPs. The above could explain the presence of TRI in the analyzed digestate samples. Moreover, according to the literature, TRI has a long half-life in organic biomass [51,52,53], which contributes to its persistence in digestate.

The concentrations of the analyzed antimicrobials were worryingly high in the studied samples, and they could induce irreversible changes in the environment by promoting the spread of AR and compromising the therapeutic efficacy of pharmaceuticals in human and veterinary medicine. According to the literature, predicted no-effect concentration (PNEC) values for resistance selection range from 8 ng/L to 64 μg/L [17], whereas in this study, drug concentrations were several or more than ten times higher. This observation gives significant cause for concern because the analyzed matrix—digestate—is often used as organic fertilizer, and therefore introduced to the natural environment.

The composition of individual digestate samples has a significant impact on the availability and degradation of antibiotics. For example, sulfadiazine and ciprofloxacin are widely used antibiotics in both human and veterinary medicine, and their residues are among the most frequently detected pharmaceuticals in the environment [6]. Sulfadiazine can be used in combination with trimethoprim in the treatment and prevention of respiratory infections and mastitis in cattle. These drugs are not fully metabolized in animals; they are excreted in feces and urine and subjected to AD in plants that process cattle slurry. Sewage sludge also increases the availability of antibiotics in the environment [8]. Wastewater treated in WWTPs contains large quantities of drugs, and contaminants such as pharmaceuticals are not fully removed during wastewater treatment, and are transferred to water bodies together with the treated wastewater [9]. In addition, the resulting sewage sludge is used as fertilizer in fields, which further increases the pool of drugs that are released into the environment. Cattle manure, sewage sludge, and digestates are widely used as fertilizers in agriculture. These potential sources of ARB, ARGs, and antibiotics exert a significant impact on agricultural ecosystems and, ultimately, on consumers of fresh vegetables or meat [5]. Digestates based on cattle slurry or sewage sludge are introduced to the soil, which is why plant substrates should also be analyzed for the presence of drugs absorbed from the applied fertilizer [1].

Furthermore, according to many authors [6,48], drugs differ in half-life and degree of biodegradation in various digestate fractions. Some drugs are biodegraded more rapidly in the solid fraction due to the accumulation of chemical compounds that favor their decomposition, whereas the compounds present in the liquid fraction are often dispersed and diluted, which may also affect their biodegradation rate. However, depending on the drug class, selected pharmaceuticals are absorbed more rapidly by the solid fraction of organic matter, where they persist for longer periods of time [16].

### 3.2. Distribution of ARGs and Integrase Genes in Digestate Samples

**Figure 2:**
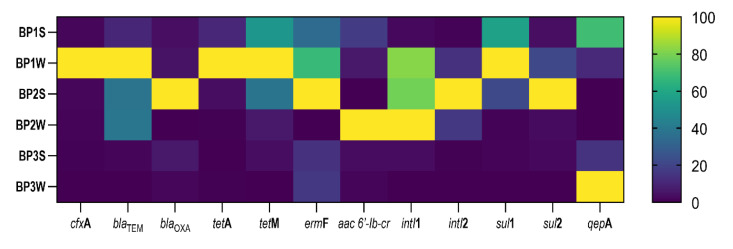
Normalized heatmap (%) presenting the absolute abundance (AA) of antibiotic resistance and integrase genes in digestate samples (number of copies/g of digestate).

As demonstrated in Figure 2, 11 ARGs conferring resistance to five antibiotic classes, as well as class 1 and 2 integrase genes, which promote the spread of AR via HGT driven by MGEs, were identified in digestate samples. Antibiotic resistance genes were detected in all digestate samples (Figure 2; Table S4, Supplementary Materials) at concentrations of 1.16 × 10^3^ to 2.25 × 10^6^ copies/g of digestate. The statistical analysis based on the Kruskal–Wallis non-parametric test did not reveal significant differences in ARG copy numbers between the analyzed samples (Kruskal–Wallis, ANOVA, *p* < 0.05) (data not shown). The abundance of ARGs was highest in sewage sludge digestate (samples BP1S and BP1W) and digestate derived from a mixture of cattle manure and maize silage (samples BP2S and BP2W).

According to Koniuszewska et al., [6] antimicrobial substances influence the prevalence of the evaluated ARGs. The presence of antibiotics in sewage sludge significantly increased (up to two orders of magnitude) the concentrations of these genes in 1 g_D_ compared with the control [16]. An increase in ARG concentrations in the digestate exposed to antimicrobials, relative to the control, could indicate that microorganisms have acquired new ARGs or have gradually adapted to the new environment [6]. According to the literature [6,16], *intI*1 and *intI*2 play an important role in the transfer of ARGs during AD. The gene *intI*1 is the most ubiquitous integrase gene in the environment which, together with sulfonamide resistance genes, has recently been recognized as an indicator of contamination with ARB, ARGs, and other anthropogenic pollutants. Integrase genes are implicated in the HGT mechanism; therefore, integron-associated genes should be included in environmental AR analyses [16]. The results of other authors are consistent with the results obtained in this study (Figure S1, Supplementary Materials), in which numerous statistically significant positive correlations between the concentration of drugs and the number of individual ARGs and integrase genes were noted.

#### 3.2.1. *tet*A and *tet*M Genes

The abundance of *tet*A and *tet*M genes encoding resistance to tetracyclines was high in the analyzed digestate samples. The abundance of the *tet*M gene ranged from 10^5^ to 10^6^ copies/g_D_, whereas the abundance of the *tet*A gene was determined at 10^4^ copies/g_D_ in most samples. The *tet*A gene was more prevalent only in sewage sludge digestate (from 10^5^ copies/g_D_ in sample BP1S to 10^6^ copies/g_D_ in sample BP1W).

The *tet* genes are ubiquitous in both Gram-negative and Gram-positive bacteria, and most bacterial *tet* genes are associated with mobile plasmids, transposons, conjugative transposons, and integrons. These genes confer tetracycline resistance via an active efflux of tetracycline (*tet*A) and by preventing bacterial ribosomes from binding to the drug (*tet*M) [54,55]. The above could explain the high concentrations of *tet* genes in the analyzed digestate samples. The high abundance of ARGs suggests that digestate-based fertilizers could pose a threat to human health. Moreover, digestate offers a supportive environment for the development of AR because it is characterized by high microbial diversity and a high content of organic carbon, nitrogen, phosphorus, and other nutrients that promote bacterial growth, even in the presence of multiple antibiotics [15].

#### 3.2.2. *sul*1 and *sul*2 Genes

The gene *sul*1 was the most abundant gene in all digestate samples, and its concentration ranged from 2.45 × 10^9^ to 8.89 × 10^10^ copies in 1 g_D_. The abundance of the *sul*2 gene was also high, but its concentration ranged from 6.25 × 10^4^ to 7.20 × 10^5^ in 1 g_D_ and was even five orders of magnitude lower in comparison with *sul*1.

Other authors also reported high concentrations of *sul*1 in cattle manure digestate [56] and *sul*2 in manure-fertilized soil [28]. Sulfonamides were the first antimicrobials to be used systemically and, according to the literature, hospital wastewater appears to be the main source of the *sul*1 gene [57]. However, sulfonamides are also widely used in the treatment and prevention of diarrhea in veterinary medicine. Sulfonamides inhibit dihydropteroate synthase in the bacterial folic acid biosynthesis pathway, and *sul*1 and *sul*2 genes confer resistance by encoding forms of dihydropteroate synthase that are not sensitive to the drug [58]. These genes can be located on transposons and mobile plasmids which confer multi-drug resistance in numerous hosts [59], which suggests that digestate-based fertilizers can promote the spread of AR in the environment.

#### 3.2.3. *aac 6′-Ib-cr* and *qep*A Genes

The abundance of the *qep*A gene ranged from 10^4^ to 10^5^ copies/g_D_ in digestate samples. The concentration of the *aac-(6′)-Ib-cr* resistance gene was one order of magnitude lower, at 10^3^ copies/g_D_ in most samples. The concentration of the *aac-(6′)-Ib-cr* gene was one order of magnitude higher (10^4^ copies/g_D_) only in sample BP1W, relative to the remaining samples. The *aac-(6′)-Ib-cr* gene encodes an enzyme which inactivates ciprofloxacin and norfloxacin by N-acetylation [60]. The *qep*A gene encodes efflux pump proteins of the major facilitator superfamily, which transport drugs to extracellular space. In the present study, the high abundance of *qep*A in digestate samples can be attributed to its non-specificity. The efflux pump encoded by *qep*A can decrease the toxic accumulation of antimicrobials inside cells, thus contributing to microbial growth during AD and promoting the transfer of ARGs between bacterial cells [61].

Other authors also reported high abundance of the *aac-(6′)-Ib-cr* gene in digestate [62], wastewater [63], and river water [64], as well as the high abundance of the *qep*A gene in sewage sludge [57]. The presence of this gene in environmental samples can be explained by the fact that it is introduced to the soil and the environment with digestate-based fertilizers. In the current study, high CIP concentrations in digestate samples suggest that this drug can increase the abundance of *aac(6′)-Ib-cr* and *qep*A genes in soil fertilized with digestate.

#### 3.2.4. *erm*F Genes

The *erm*F gene was abundant in all digestate samples, regardless of their origin, and its concentration ranged from 10^7^ to 10^8^ copies/g_D_. The *erm*F gene was most abundant (10^8^ copies/g_D_) in samples of cattle manure digestate. These results suggest that digestate is a hot spot of ARGs. In the literature, strains of *Enterococcus* resistant to erythromycin were identified in ready-to-eat meat products, which implies that these genes can be transmitted to the gut microbiota of livestock and animal-based foods [65].

#### 3.2.5. *cfx*A, *bla*_TEM_ and *bla*_OXA_ Genes

The gene *cfx*A was the most abundant beta-lactam-resistance gene, and its concentration reached 3.33 × 10^5^ copies/g of digestate in sample BP1W (sewage sludge digestate).

All beta-lactam-resistance genes were also identified in digestate samples from BP3, which processes maize silage, slaughterhouse waste, potato pulp, and confectionary press cake (samples BP3S and BP3W), but their concentrations were one order of magnitude lower on average (10^3^ to 10^4^ copies/g_D_) than in the remaining samples (10^5^ copies/g_D_). Genes encoding resistance to beta-lactams are ubiquitous in the environment. The analyzed digestate samples were free of beta-lactams that could exert selection pressure, but they were characterized by high concentrations of beta-lactam-resistance genes that can be transported the soil environment with digestate, accumulate in plants and, consequently, reach the animals’ gut microbiota [28,66].

#### 3.2.6. *intI*1 and *intI*2 Genes

Integrons participate in the spread of ARGs via HGT mediated by MGEs, and the *intI*1 gene is regarded as an indicator of HGT [67]. In the present study, both integrase genes (*intI*1 and *intI*2) were abundant in all digestate samples (regardless of their substrate composition), and their concentrations ranged from 4.37 × 10^5^ to 5.84 × 10^7^ copies/g_D_. These results could indicate that MGEs are involved in the spread of AR.

Class 1 and 2 integrons (*intI*1, *intI*2) were the first integrons to be strongly associated with MGEs. In other studies, sewage sludge and manure digestates frequently harbored *intI*1 [68,69] and *intI*2 genes [70]. There is evidence to suggest that digestate-based fertilizers containing integrase genes contribute to the transmission of ARGs [71] and accelerate the spread of AR with the involvement of MGEs [72].

In summary, the results of this study indicate that digestate contaminated with ARGs can lead to the development of antibiotic resistance in soil bacteria. Antibiotic resistance genes are transported to the soil environment by digestate and are accumulated by host bacteria in soil. They can spread via HGT or novel mutations in soil bacteria [73]. Moreover, ARGs can spread even if antibiotics have been completely or partially degraded [74]. This is due to the fact that other environmental contaminants [75], such as HMs [76], can also co-select for resistance genes and contribute to the persistence of ARGs in the environment [77].

### 3.3. Heavy Metals in Digestate Samples

The presence and concentrations of the analyzed HMs differed significantly (ANOVA, Kruskal–Wallis, *p* < 0.05) in digestate samples, depending on their origin. Heavy metals were identified in all samples, but not every digestate sample contained all of the analyzed HMs. Heavy metals such as Cd, Ni, Cr, Zn, and Hg were present in all samples. Lead was detected in sewage sludge digestate and digested mixtures of cattle manure and maize silage, but not in digestate originating from green waste. Sewage sludge digestate and digested mixtures of cattle manure and maize silage were characterized by similar HM levels (Table 2). Zinc was the most abundant HM, and its concentration ranged from 595 mg/kg _dm_ in sample BP2W (digested cattle manure and maize silage) to 830 mg/kg _dm_ in sample BP1S.

Relatively high Zn concentrations in digested cattle manure and maize silage can probably be attributed to growth-promoting feed additives with increased levels of Cu and Zn. Zinc induces changes in the gut microbiome and inhibits anaerobic fermentation in the intestines, which decreases the availability of nutrients for fermenting bacteria and suppresses the growth of gut pathogens. Zinc is poorly absorbed in the intestines; it is excreted with feces and transferred to BPs that digest manure. Zinc and other HMs can accumulate in soils that are frequently fertilized with digestate [78]. In other studies, high concentrations of HMs were also reported in manure [28], agricultural soil [79], sewage sludge [80], and aquatic environments [81], which suggests that in addition to antibiotics, toxic HMs such as Hg, Cu, and Zn can exert strong selection pressure and increase ARG levels in the environment [28,78].

Heavy metal pollution from the livestock sector could promote the spread of AR by co-selection. Therefore, bacterial communities colonizing agricultural soils are strongly exposed to a combination of HMs and antibiotics. Exposure to both antimicrobial substances may increase the likelihood of selection and co-selection of AR [28]. The co-occurrence of HM resistance genes and ARGs on the same MGEs, such as integrons, is referred to as co-resistance. Co-resistance significantly facilitates the spread and proliferation of resistance in microbial communities. Cross-resistance is yet another mechanism responsible for the co-selection of bacterial resistance to antimicrobials and HMs. In cross-resistance, antibiotics and HMs initiate the same biochemical pathways, leading to the emergence of HM-driven AR co-regulation as the final mechanism in the co-selection of resistance to drugs and HMs. In this process, high concentrations of HMs in the environment increase bacteria’s resistance to antibiotics by regulating the expression of specific genes, thus reducing microbial susceptibility to these compounds [16].

### 3.4. Statistical Analysis

#### Principal Component Analysis (PCA) of ARGs, Pharmaceuticals and HMs

**Figure 3:**
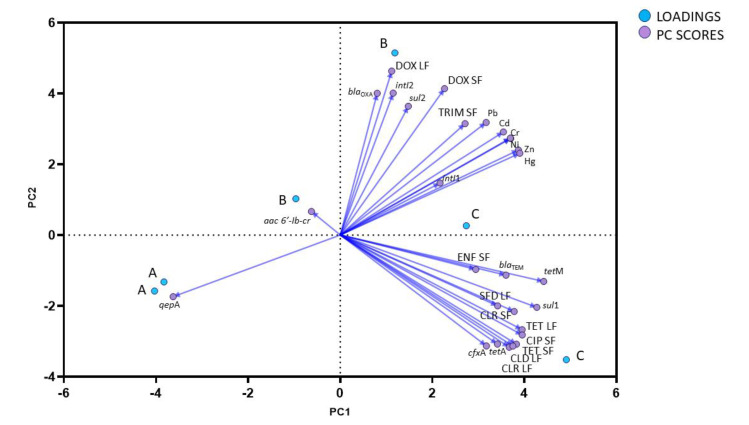
Principal Component Analysis (PCA) based on the distribution of ARGs and integrase genes, pharmaceuticals, and heavy metals (HMs) in all investigated samples. Mean loadings for ARGs and the content of antibiotics and HMs, and mean PC scores for biogas plants: A—biogas plant 1 (BP1), B—biogas plant 2 (BP2) and C—biogas plant 3 (BP3).

Principal component analysis was used using all data from all samplings as variables to assess the overall patterns and variances of antibiotic concentrations, ARG, and HM levels in the BP digestate samples. The PCA produced three main data clusters (Figure 3). On the PCA-presented mean loadings for ARGs, the content of antibiotics and HMs, and mean PC scores for biogas plants: A—biogas plant 1 (BP1), B—biogas plant 2 (BP2) and C—biogas plant 3 (BP3). The results for BP2 (B) revealed strong correlations between the values of DOX, *bla*_OXA_, *sul*2, *intI*1, *intI*2, HMs, and TRI. The second cluster was formed by BP1, with strong correlations between the values of ENF, SFD, CLR, CLD, CIP, TC, *cfx*A, *tet*A, *tet*M, *sul*1, and *bla*_TEM_. However, strong correlations with HM values were not observed. The values noted in each cluster suggest that resistance-inducing antibiotics, ARGs, and HMs had a common source. It can be concluded that the biogas plant processing both sewage sludge and liquid manure is a reservoir of drugs, ARGs, and HMs. The results of the PCA also indicate, with high probability, that HMs could contribute to an increase in ARG concentrations in the BP processing cattle manure (BP2) and promote the spread of AR. According to previous research [82], the presence of strong correlations between ARGs in digestate suggests that ARB could be present already in raw substrates. In the present study, the antibiotics detected in digestate samples could have exerted selection pressure during the AD process.

The results of the PCA suggest that soil fertilization with digestate from agricultural BPs can increase the abundance of various ARGs, metal resistance genes (MRGs), drugs, drug residues and the associated microorganisms in the soil environment, and that digestate can significantly contribute to the spread of ARGs in the environment.

### 3.5. Implications for the Environment

Digestate is a rich source of organic matter and nutrients, and it is often used as organic fertilizer. However, digestate is also a vector of veterinary antibiotics, which are transmitted to the soil ecosystem and plants [17] because the spread of antibiotics and ARGs in the environment is not limited to a single ecosystem. Antibiotics are transported between all ecological niches that form a specific network [83] which also carries ARGs, leading to artificial selection of microorganisms that initially are not resistant to antibiotics. Selection pressure from human activities and antibiotics influences evolutionary processes in the environment. A strong phylogenetic relationship was observed between human pathogens and pathogens colonizing livestock [84]. The spread of ARGs in the food chain can undermine the efficacy of antibiotics for treating infectious diseases in both livestock and clinic and hospital patients. The wide spread of ARGs in the environment gives serious cause for concern. Another study demonstrated that manure storage in the proximity of animal pens increased resistance to chlortetracycline and tetracycline in soil bacteria [85]. Manure and digestate storage sites are permanent reservoirs of ARB and ARGs.

Abiotic factors can also contribute to the spread of ARGs. Physical factors such as wind, surface runoffs, flowing river water, and human activity can also increase AR in pathogenic bacteria in the environment [16,86,87,88]. Animals are raised on farms in close proximity to humans, and antibiotics are widely used as growth promoters to increase productivity and cater to the nutritional demands of the world’s rapidly growing population, which increases the risk of ARGs transferring to the animals’ gut microbiota. The substrates processed in agricultural BPs, including manure, green waste, and sewage sludge, are reservoirs of antibiotics and ARGs. The present study demonstrated that antimicrobials present in digestate samples in microgram quantities can be transferred to the environment and promote the evolution of bacteria able to grow at drug concentrations as high as 1000 mg/L. These results are comparable with the PNEC values estimated by some authors [89,90]. Drugs can significantly influence bacterial growth, the structure and composition of soil microbial communities, soil quality, and the quality of crops contaminated with high concentrations of antibiotics and ARGs [91].

Despite the fact that digestate is the end product of the AD process, the analyzed digestate samples were characterized by high concentrations of ARGs and antibiotics. These observations suggest that digestate-based fertilizers should be applied at intervals longer than two months to restore background levels of ARGs in digestate-modified soils. This strategy would improve agricultural practices, inhibit the spread of ARGs, and decrease their concentrations in the environment [92,93,94,95].

Effective strategies for controlling the use of antibiotics in agriculture are the simplest solution to reducing the concentrations of drugs and ARGs in cattle slurry, AD substrates, digestates, and farmland. Such strategies should be urgently implemented to minimize the health risks and adverse economic impacts of agricultural products containing drugs, ARB, and ARGs. Bioremediation and microbial electrolysis techniques are promoted in the agricultural sector to remove antibiotic residues and reduce AR. Heavy metals can be extracted from soil or wastewater with kaolin and sulfate-reducing bacteria. An integrated global strategy is needed to control the use of antibiotics in hospitals and the livestock sector, and novel methods should be introduced to optimize wastewater treatment, composting, and bioremediation of soil pollutants.

## 4. Conclusions

Studies evaluating the presence and concentrations of various antibiotic classes and HMs in complex matrices, such as digested cattle manure, maize silage, sewage sludge, slaughterhouse waste, green waste, and confectionary press cake, pose an analytical challenge because different protocols have to be applied depending on digestate fraction and composition. Moreover, the composition of digestate samples can vary considerably because many agricultural BPs process mixed substrates with different proportions of plant biomass and manure. Industrial residues such as slaughterhouse waste also pose a considerable challenge for researchers. Despite these problems, this study demonstrated that most of the analyzed antibiotics and HMs were not degraded during the AD process. Digestate samples were highly contaminated with various drugs and HMs whose concentrations were influenced by the composition of the substrates processed by the examined BPs. Attention should also be paid to factors that contribute to the spread of AR in plant substrates. Unlike sewage sludge and cattle manure digestates, plant-based digestates did not contain drugs, but were characterized by high concentrations of many ARGs and selected HMs. Digestate can be regarded as a reservoir of ARGs due to the high abundance of various ARGs. Digestate samples were free of beta-lactams, but contained numerous genes encoding resistance to this class of antibiotics, which could point to high levels of AR already in the substrates. High concentrations of integrase genes also suggest that MGEs participate in the transmission of AR in digestate. The results of this study indicate that the use of digestate as organic fertilizer increases the risk of soil contamination with antibiotics, HMs, and ARGs.

## Figures and Tables

**Table 1:** Characteristics and composition of the examined digestate samples.

Sample	Composition of Substrates	pH	Dry Mass (%)	FOS/ TAC Ratio	NH_4_+ g/L	TN g/L	TP g/L
BP1S	Sewage sludge	8.40	14.9	0.15	1.10	6.50	2.80
BP1W		7.62	16.00	0.17	1.16	6.50	2.46
BP2S	Cattle manure, maize silage	6.70	3.01	0.19	1.20	na	3.00
BP2W		6.88	3.00	0.14	1.43	na	3.65
BP3S	Maize silage, slaughterhouse waste, potato pulp, confectionery press cake	7.83	4.80	0.18	2.40	na	na
BP3W		7.90	4.30	0.14	1.60	na	na

na—not available.

**Table 2:** Content of heavy metals (HMs) in digestate samples.

	Digestate Samples					
HMs (mg/kg_dm_)	BP1S	BP1W	BP2S	BP2W	BP3S	BP3W
Cd	1.60	0.90	1.60	0.90	0.02	0.03
Pb	18.00	7.13	20.00	7.90	0.00	0.00
Ni	17.00	11.00	19.00	12.00	0.37	0.32
Cr	44.00	28.00	40.00	26.00	0.2	0.25
Zn	830.00	645.00	790.00	595.00	10.40	11.90
Hg	0.51	0.4144	0.49	0.3112	0.0097	0.0037

HMs—heavy metals; dm—dry mass; Cd—cadmium; Pb—lead; Ni—nickel; Cr—chromium; Zn—zinc; Hg—mercury.

## Data Availability

Not applicable.

## Supplementary Materials

The following supporting information can be downloaded at: https://www.mdpi.com/article/10.3390/ijerph20032672/s1, Table S1: Procedures used in the determination of antimicrobials in digestate samples; Table S2: Parameters of the qPCR reaction; Table S3: Antibiotic concentrations in digestate samples; Table S4: The presence and absolute abundance of antibiotic resistance genes (ARGs) in digestate samples (abbreviations are explained in Table S3 (number of copies/g of digestate); Figure S1: Spearman’s non-parametric correlation analysis between antibiotic concentrations and concentrations of ARGs, integrase genes, physicochemical parameters (TN–total nitrogen; TP–total phosphorus), and concentration of HMs (Cd-cadmium, Pb–lead, Ni–nickel, Cr–chromium, Zn–zinc, and Hg–mercury) (other abbreviations are explained in Table S1 and Table S3). Sig-nificant statistical results are marked with an asterisk: * for *p* < 0.05; ** for *p* < 0.01; *** *p* < 0.001).
