# Supplementary material for: Digestate from Agricultural Biogas Plants as a Reservoir of Antimicrobials and Antibiotic Resistance Genes—Implications for the Environment
Source: Int J Environ Res Public Health. 2023 Feb 2;20(3):2672. doi: 10.3390/ijerph20032672 (PMC9915926; doi:10.3390/ijerph20032672)
Supplement: Supplementary file 1 [file ijerph-20-02672-s001.zip › ijerph-2147024-supplementary.pdf]

Supplementary Materials

Digestate from agricultural biogas plants as a reservoir of antimicrobials and antibiotic resistance genes – implications for the environment

Izabela Wolak[1], Sylwia Bajkacz[2], Monika Harnisz[1], Klaudia Stando[2], Magdalena Męcik[1], Ewa Korzeniewska[1*]

[1] Department of Water Protection Engineering and Environmental Microbiology, Faculty of Geoengineering, University of Warmia and Mazury in Olsztyn, Prawocheńskiego 1, 10-720 Olsztyn, Poland
[2] Department of Environmental Biotechnology, Faculty of Energy and Environmental Engineering, Silesian University of Technology, Akademicka 2, 44-100, Gliwice, Poland
* Correspondence: ewa.korzeniewska@uwm.edu.pl

Table S1. Procedures used in the determination of antimicrobials in digestate samples.

Class of antimicrobials	Compound	Fraction	References
betalactams	AMO	S	[1]
		L	[2]
	AMP	S	[1]
		L	[2]
tetracyclines	TC	S	[1]
		L	[2]
	OXY	S	[1]
		L	[2]
	DOX	S	[1]
		L	[2]
	CHLOR	S	[1]
		L	[2]
sulphonamides	SMX	S	[1]
		L	[2]
	SFD	S	[3]
		L	[2]
fluoroquinolones	ENF	S	[1]
		L	[2]
	CIP	S	[1]
		L	[2]
macrolides	CLR	S	[3]
		L	[2]
lincosamides	CLD	S	[3]
		L	[2]
dihydrofolic acid reductase inhibitor	TRI	S	[3]
		L	[2]

AMO – amoxicillin ; AMP – ampicillin; TC – tetracycline; OXY – oxytetracycline; DOX – doxycycline; CHLOR – chlortetracycline; SMX – sulfamethoxazole; SFD – sulphonamide; ENF – enrofloxacin; CIP – ciprofloxacin; CLR – clarithromycin ; CLD – clindamycin; TRI – trimethoprim; S – solid fraction; L – liquid fraction

Table S2. Parameters of the qPCR reaction.

Gene	Primer (5′–3′)	Amplicon size (bp)	Annealing temperature (°C)	Reference

*cfx*A	CGTAGTTTTGAGTATAGCTTT<br>GATGTTGCCTATATATGTC	802	56	[1]
bla$_{\text{TEM}}$	AGTGCTGCCATAACCATGAGTG<br>CTGACTCCCCGTCGTGTAGATA	431	61	[2]
bla$_{\text{OXA}}$	ATTATCTACAGCAGCGCCAGTG<br>TGCATCCACGTCTTTGGTG	296	61	
*tet*A	GCTACATCCTGCTTGCCTTC<br>GCATAGATCGCCGTGAAGAG	211	54	[3]
*tet*M	ACAGAAAGCTTATTATATAAC<br>TGGCGTGTCTATGATGTTCAC	171	55	
aac 6′-Ib-cr	TTGCAGTGCTCTATGAGTGGCTA<br>CTCGAATGCCTGGCGTGTTT	482	57	[4]
*qep*A	GCCGGTGATGCTGCTGA<br>CAGTAACAGCGCACCAA	204	63	[5]
*sul*1	CGCACCGGAAACATCGCTGCAC<br>TGAAGTTCCGCCGCAAGGCTCG	163	55.9	[4]
*sul*2	TCCGGTGGAGGCCGGTATCTGG<br>CGGGAATGCCATCTGCCTTGAG	191	57.5	
*int*I1	CCTCCCGCACGATGATC<br>TCCACGCATCGTCAGGC	280	54	[5]
*int*I2	TTATTGCTGGGATTAGGC<br>ACGGCTACCCTCTGTTATC	233	50	
*erm*F	TAGATATTGGGGCAGGCAAG<br>GGAAATTGCGGAACTGCAAA	126	58	[6]

Table S3. Antibiotic concentrations in digestate samples.

			**Samples of digestate from biogas plants**					
Antibiotic	Fraction	Parameters	BP1W	BP1S	BP2W	BP2S	BP3W	BP3S
SFD	LF	Av.	136.5	98.2	106.8	n.d	n.d	n.d
		SD.	21.6	14.3	8.8	-	-	-
	SF	Av.	n.d	n.d	n.d	n.d	n.d	n.d
		SD	-	-	-	-	-	-
TC	LF	Av.	368.7	275.6	n.d	n.d	n.d	n.d
		SD	27.2	32.3	-	-	-	-
	SF	Av.	1164.4	464.8	n.d	n.d	n.d	n.d
		SD	216.3	104.8	-	-	-	-
DOX	LF	Av.	<LOQ	620.4	854.6	1555.9	n.d	n.d
		SD	-	34.2	142.7	305.9	-	-
	SF	Av.	218.1	800.2	396.7	1282.5	n.d	n.d
		SD	26.4	131.1	69.5	193.8	-	-
CIP	LF	Av.	n.d	n.d	n.d	n.d	n.d	n.d
		SD	-	-	-	-	-	-
	SF	Av.	145.3	92.4	n.d	n.d	n.d	n.d
		SD	21.1	13.4	-	-	-	-
ENF	LF	Av.	n.d	n.d	n.d	n.d	n.d	n.d
		SD	-	-	-	-	-	-
	SF	Av.	147.1	387.9	n.d	n.d	n.d	n.d
		SD	21.8	56.2	-	-	-	-
CLR	LF	Av.	208.9	56.97	n.d	n.d	n.d	n.d
		SD	27.2	8.3	-	-	-	-

	SF	Av.	62.8	70.7	n.d	n.d	n.d	n.d
		SD	9.8	9.4	-	-	-	-
CLD	LF	Av.	268.4	88.6	n.d	n.d	n.d	n.d
		SD	29.1	8.7	-	-	-	-
	SF	Av.	n.d	n.d	n.d	n.d	n.d	n.d
		SD	-	-	-	-	-	-
TRI	LF	Av.	n.d	n.d	n.d	n.d	n.d	n.d
		SD	-	-	-	-	-	-
	SF	Av.	66.8	226.1	<LOQ	261.9	n.d	n.d
		SD	7.6	42.5	-	53.2	-	-

BP – biogas plant; 1,2,3 – Number of BP; W – winter; S – spring; SFD - sulfadiazine; TC - tetracycline; DOX - doxycycline; CIP - ciprofloxacin; ENF - enrofloxacin; CLR - clarithromycin; CLD - clindamycin; TRI - trimethoprim; Av. – Average (ng/g in solid fraction or ng/L In liquid fraction); SD – standard deviation; n.d – not detected;

Table S4. The presence and absolute abundance of antibiotic resistance genes (ARGs) in digestate samples (abbreviations are explained in Table S3) (number of copies/g of digestate).

	**Targeted genes and class of antibiotics**											
	betalactams			tetracyclines		fluoroquinolones		sulfonamides		MLS group	Integrase genes	
Sample	*cfx*A	bla_{TEM}	bla_{OXA}	*tet*A	*tet*M	*aac* 6′-Ib-cr	*qep*A	*sul*1	*sul*2	*erm*F	*int*I1	*int*I2
BP1S	4.98E3	1.07E4	2.60E3	2.47E5	1.26E6	9.50E3	2.34E5	5.19E10	1.63E5	4.13E7	4.37E5	4.37E5
BP1W	3.33E5	7.78E4	3.05E3	2.22E6	2.25E6	5.02E3	5.10E4	8.89E10	7.2E5	7.08E7	7.24E6	8.14E6
BP2S	4.98E3	3.11E4	4.25E4	7.87E4	9.65E5	2.60E3	1.60E4	2.11E10	3.14E6	1.00E8	6.94E6	5.84E7
BP2W	2.79E3	3.21E4	1.16E3	1.28E4	2.89E5	4.25E4	1.60E4	2.9E9	1.42E5	1.06E7	8.76E6	9.39E6
BP3S	1.50E3	3.46E3	3.70E3	1.10E4	2.29E5	3.70E3	6.15E4	2.98E9	1.19E5	2.30E7	5.19E5	1.39E5
BP3W	5.73E2	2.83E3	1.67E3	1.21E4	1.64E5	3.05E3	3.31E5	2.45E9	6.25E4	2.47E7	2.78E5	1.11E5

S – spring; W – winter

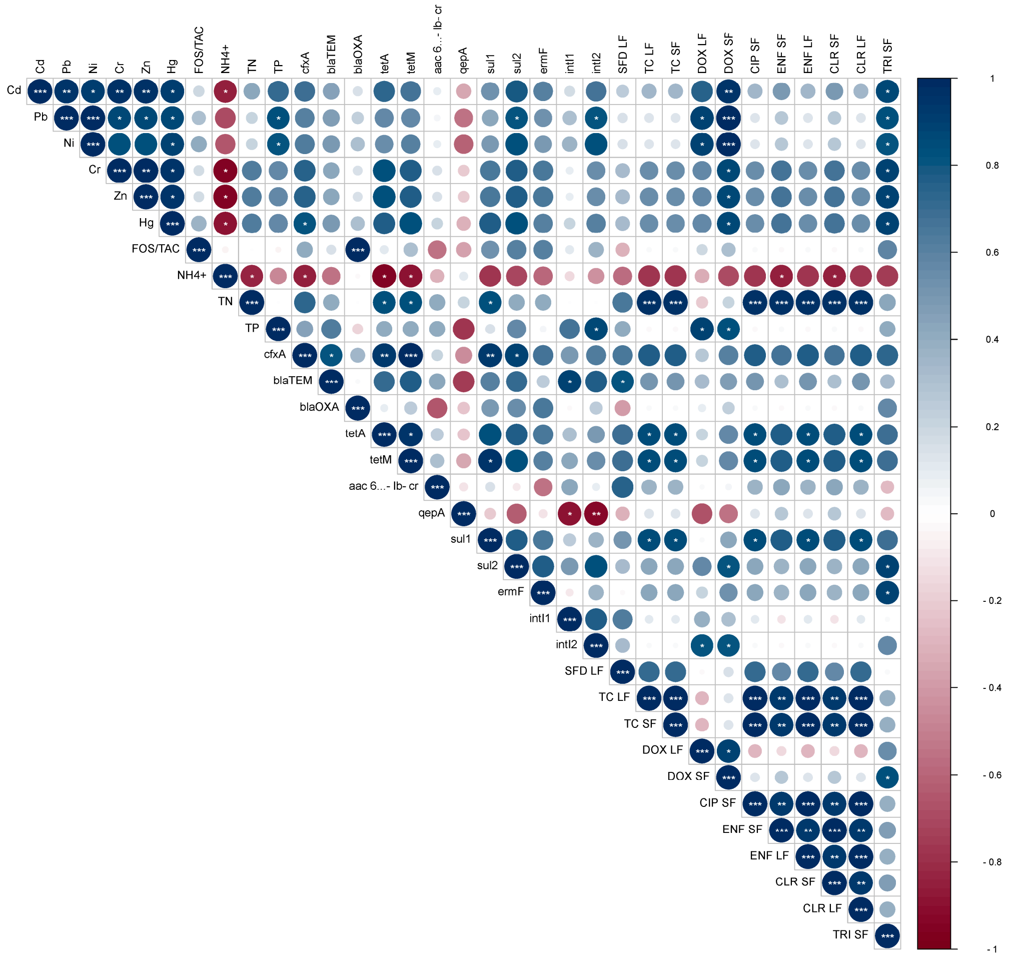

Figure S1. Spearman's non-parametric correlation analysis between antibiotic concentrations and concentrations of ARGs, integrase genes, physicochemical parameters (TN – total nitrogen; TP – total phosphorus), and concentration of HMs (Cd - cadmium, Pb – lead, Ni – nickel, Cr – chromium, Zn – zinc, and Hg – mercury) (other abbreviations are explained in Table S1 and Table S3). Significant statistical results are marked with an asterisk: * for p<0,05; ** for p<0.01; *** p<0.001).

References

1. Bajkacz, S.; Felis, E.; Kycia-Słocka, E.; Harnisz, M.; Korzeniewska, E. Development of a new SLE-SPE-HPLC-MS/MS method for the determination of selected antibiotics and their transformation products in anthropogenically altered solid environmental matrices. Sci. Total Environ. 2020, 726, doi:10.1016/j.scitotenv.2020.138071.
2. Kokoszka, K.; Zieliński, W.; Korzeniewska, E.; Felis, E.; Harnisz, M.; Bajkacz, S. Suspect screening of antimicrobial agents transformation products in environmental samples development of LC-QTrap method running in pseudo MRM transitions. Sci. Total Environ. 2022, 808, doi:10.1016/j.scitotenv.2021.152114.
3. Buta, M.; Korzeniewska, E.; Harnisz, M.; Hubeny, J.; Zieliński, W.; Rolbiecki, D.; Bajkacz, S.; Felis, E.; Kokoszka, K. Microbial and chemical pollutants on the manure-crops pathway in the perspective of "One Health" holistic approach. Sci. Total Environ. 2021, 785, doi:10.1016/j.scitotenv.2021.147411.
4. Pei, R.; Kim, S.C.; Carlson, K.H.; Pruden, A. Effect of River Landscape on the sediment concentrations of antibiotics and corresponding antibiotic resistance genes (ARG). Water Res. 2006, 40, 2427–2435, doi:10.1016/j.watres.2006.04.017.
5. Goldstein, C.; Lee, M.D.; Sanchez, S.; Hudson, C.; Phillips, B.; Register, B.; Grady, M.; Liebert, C.; Summers, A.O.; White, D.G.; et al. Incidence of class 1 and 2 integrases in clinical and commensal bacteria from livestock, companion animals, and exotics. Antimicrob. Agents Chemother. 2001, 45, 723–726, doi:10.1128/AAC.45.3.723-726.2001.
6. Eitel, Z.; Sóki, J.; Urbán, E.; Nagy, E. The prevalence of antibiotic resistance genes in Bacteroides fragilis group strains isolated in different European countries. Anaerobe 2013, 21, 43–49, doi:10.1016/j.anaerobe.2013.03.001.
